# Second- and Third-Tier Therapies for Severe Traumatic Brain Injury

**DOI:** 10.3390/jcm11164790

**Published:** 2022-08-16

**Authors:** Charikleia S. Vrettou, Spyros D. Mentzelopoulos

**Affiliations:** First Department of Intensive Care Medicine, Evaggelismos General Hospital, National and Kapodistrian University of Athens Medical School, 10676 Athens, Greece

**Keywords:** brain trauma, intracranial hypertension, neuromonitoring

## Abstract

Intracranial hypertension is a common finding in patients with severe traumatic brain injury. These patients need treatment in the intensive care unit, where intracranial pressure monitoring and, whenever possible, multimodal neuromonitoring can be applied. A three-tier approach is suggested in current recommendations, in which higher-tier therapies have more significant side effects. In this review, we explain the rationale for this approach, and analyze the benefits and risks of each therapeutic modality. Finally, we discuss, based on the most recent recommendations, how this approach can be adapted in low- and middle-income countries, where available resources are limited.

## 1. Introduction

Traumatic brain injury (TBI) is defined as “an alteration in brain function, or other evidence of brain pathology caused by an external force”, while severe TBI is specified by a score of ≤8 in the Glasgow Coma Scale (GCS) [1]. Severe TBI is a multifaceted condition, rather than a single disease. According to a recent, large, prospective study, the most common mechanisms for severe TBI are falls in very-high-income countries, and road traffic accidents elsewhere [2]. Pathological findings may be focal or global. Focal findings include extradural, subdural and intracerebral hematomas, as well as contusions. Global findings include diffuse axonal injury, brain swelling and ischemia, and post-traumatic hydrocephalus [3]. A common characteristic of severe TBI is intracranial hypertension (ICH), which ranges, in different cohorts, from 50 to 80% of the cases [4].

ICH is considered an important component of TBI pathophysiology because it is related to significant morbidity and mortality, and carries the risk of cerebral herniation [5,6,7]. The latest recommendations by the Brain Trauma Foundation advise to maintain intracranial pressure (ICP) at ≤22 mmHg and cerebral perfusion pressure (CPP) at ≥60 mmHg. An escalating approach has been adopted for the treatment of ICH [3,8,9,10,11,12,13], according to which different treatment modalities are prioritized on the basis of their efficacy and relative risks of their application [8,9,11,13,14,15]. Treatments with more severe side effects are classified as higher tier, while safer treatments, such as analgesia, sedation, and hyperosmolar therapy, are considered lower tier. It is expected that refractory ICH will eventually require higher-tier therapies that carry higher risk of complications. The concept of separate tiers serves two important purposes. Firstly, it alerts physicians to the increased risks associated with escalation of treatment. Before taking those risks, it is advised that a thorough examination and repeated imaging be performed, as appropriate. Medical imaging, in particular computerized tomography (CT) of the brain, may reveal evolving lesions, such as contusions and hematomas that demand surgical evacuation, rather than escalation of medical treatment [8,10,11,12,15,16,17]. A detailed medical and neurological evaluation, on the other hand, may identify extracranial causes of ICP elevation, such as infections, respiratory deterioration, and sodium disturbances [3,8,9,10,11,12,18,19]. Secondly, a tiered design allows for flexible choices among the modalities of each tier, or even for skipping a tier, if considered advantageous to the patient. In order to apply the tier concept effectively, clinicians need to be familiar with the treatments involved and to be aware of their side effects. They also need to formulate a treatment and escalation strategy that is tailored to the clinical presentation and therapeutic needs of each patient.

**Figure 1:**
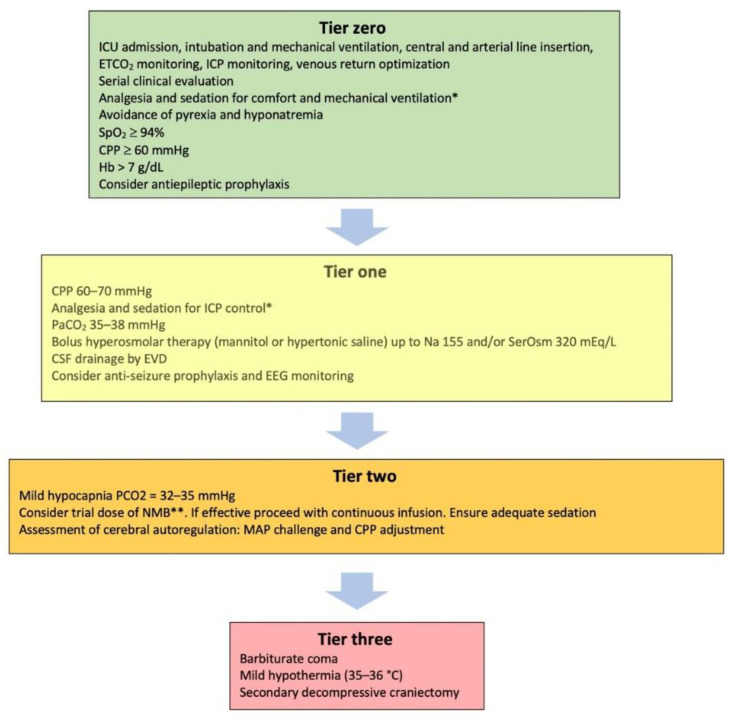
Treatment modalities included in the tiered approach for the management of intracranial hypertension. ICU: intensive care unit; ETCO_2_: end-tidal carbon dioxide partial pressure; ICP: intracranial pressure; SpO_2_: oxygen saturation; CPP: cerebral perfusion pressure; Hb: hemoglobin concentration; SerOsm: serum osmolality; CSF: cerebrospinal fluid; EVD: external ventricular drain; EEG: electroencephalography; NMB: neuromuscular blocker; MAP: mean arterial pressure. * Propofol and midazolam are the most commonly used anesthetic agents. Morphine, fentanyl, sulfentanil and remifentanil are the most commonly used analgesics [20]. ** Non-depolarizing agents are considered as safer than succinylcholine [21].

The purpose of this review is to focus on treatments that carry significant side effects, the so called second- and third-tier therapies [8] in patients with blunt traumatic brain injury. A prerequisite for the safe management of these patients is their admission to the intensive care unit (ICU), where the required interventions can be applied in the safest possible way. Basic support measures applied in the ICU are considered tier zero therapies, while initial treatments targeting the ICH are classified into tier one (Figure 1) [8]. 

As shown in Figure 1, tier one therapies targeting at lowering the ICP include escalation of analgesia and sedation, normocapnia, the use of an external ventricular drain (EVD) to drain cerebrospinal fluid (CSF), and hyperosmolar therapy. Both mannitol 20% and hypertonic sodium chloride solutions can be used as hyperosmolar agents. Common side effects include derangements of the fluid and electrolyte balance, and for this reason, ICP- guided (rather than scheduled) administration is advised [22]. In cases with significant brain edema, the placement of an EVD can be technically difficult, with higher risk of hemorrhage and misplacement, because the ventricular system may be compressed [23]. Furthermore, the presence of an EVD carries a small but significant risk of infection [24]. Even though tier one interventions are not free of complications, treatments beyond this level have recently been related to an additional negative effect on survival [14,19]. For this reason, tier-two and -three therapies require increased caution and clinical experience for their safe application [15].

## 2. Tier-Two Therapies

### 2.1. Mild Hypocapnia (PaCO_2_ 32–35 mmHg)

Mild hypocapnia, targeting a PaCO_2_ of 32–35 mmHg, is among the second-tier choices. Mild hyperventilation is an effective and rapid way to reduce the ICP by inducing cerebral vasoconstriction and reducing cerebral blood flow [25]. However, it carries the risk of cerebral ischemia. A study in brain injured patients and normal controls, using multimodal neuromonitoring and positron emission tomography (PET) of the brain, showed that mild hypocapnia significantly reduced the ICP, but also increased the volume of ischemic brain tissue in both perilesional and normal regions of the brain, compared to normocapnia levels and to normal controls [26]. Interestingly, ischemic brain volume increased even when jugular venous saturation (SjO_2_) measurements, used for the assessment of global brain oxygen demands, were within the acceptable range. More recent studies report acceptable cerebral oxygenation and blood flow parameters during mild hyperventilation [27,28,29,30]. A practical approach, based on current evidence, is the application of mild hyperventilation with concomitant use of multimodal neuromonitoring, including methods for the focal and global assessment of the cerebral oxygenation adequacy [8,10,31]. Such modalities are the perfusion computerized tomography (CTP), the transcranial color duplex ultrasonography (TCD), as well as the SjO_2_ and the brain parenchymal oxygenation (PbrO_2_) monitoring. In practice, these techniques are not always available in general ICUs, thus limiting the application of mild hypocapnia as a potentially useful intervention. In view of such restrictions, and based on the current limited evidence, mild hyperventilation is considered an acceptable measure before escalating to higher tiers. In any case, close monitoring of the PaCO_2_ is important to avoid an accidental reduction in the PaCO_2_ below 30 mmHg [32].

**Figure 2:**
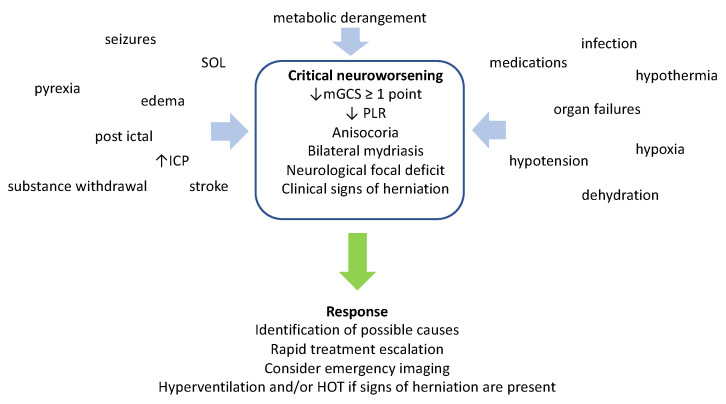
Definition, causes, and management of critical neuroworsening. mGCS: motor Glasgow Coma Score; PLR: pupillary light reflex; SOL: space-occupying lesion; ICP: intracranial pressure; HOT: hyperosmolar therapy.

In addition to the risk of brain ischemia, lowering the PCO_2_ may pose additional problems to trauma patients. The subsequent rise of the pH carries the risk of reduced blood flow to other body organs and has been related to tissue hypoxia, cardiac arrythmias, hypokalemia, hypocalcemia, hypophosphatemia, and lower epileptic threshold [31]. Moreover, increased tidal volumes may be necessary for mild hypocapnia, and this may render mechanical ventilation traumatizing to the lung [33]. Since blood flow to the brain is known to be reduced during the first 24 h after brain trauma, it is reasonable to avoid mild hypocapnia during this time period [34]. Finally, hypocapnia below 30 mmHg had better be kept as a temporizing measure for cases of extremes in ICH, when signs of critical neuroworsening (Figure 2) or impending herniation, such as the Cushing’s reflex (i.e., increased blood pressure, bradycardia, and irregular breathing if the patient is not already deeply sedated and mechanically ventilated) are present. Acute hypocapnia can be applied as a bridge to higher-tier therapies, for a limited period of time, until other, longer acting measures are in place. Subsequently, a gradual return to normocapnia is advised, in order to avoid rebound ICH [11].

### 2.2. Neuromuscular Blockade (NMB)

Neuromuscular blockers, mostly non-depolarizing ones, such as cis-atracurium, have been used in the past as a tier zero therapy in patients with brain trauma [14]. There is, however, limited evidence for the effect of NMB on ICH. It has been described that paralysis may lower the ICP by 2–3 mmHg [21]. Theoretically, lowering ventilation pressures and limiting ventilator asynchrony can improve venous outflow to the main vessels of the chest [35]. Even though this has a likely beneficial effect, NMB should be applied with caution, and for short periods.

In one study that included TBI patients, increased rates of ventilator associated pneumonia and prolonged ICU stay was reported in patients who received NMB for >12 h compared to those who did not [36]. Another concern is the effect of NMB on the long-term outcomes. Prolonged use of NMB is associated with ICU-acquired neuromuscular weakness, also known as ICU neuromyopathy [37,38]. This entity can significantly affect the quality of life of the patients and is related to the post-intensive care syndrome, which affects more than 60% of ICU survivors [39]. Prolonged immobilization, steroid and aminoglycoside use have also been shown to contribute to ICU neuromyopathy [40,41].

NMB may be indicated for trauma patients who, in addition to brain injury, also have lung contusions, acute respiratory distress syndrome (ARDS) or abdominal compartment syndrome [35,42]. NMB is also justified during stimulating procedures, such as tracheal suction and bronchoscopy, in patients who are deeply sedated [43], and can be necessary during the application of cooling measures to lower body temperature. Since muscular activity significantly contributes to CO_2_ production, NMB can assist CO_2_ control [44]. A trial for NMB is currently suggested for patients in whom ICH is not controlled with tier one measures, with continued infusion reserved for those who show a favorable response, or for patients who need NMB for other reasons, as previously described [21].

### 2.3. Assessment of Static Autoregulation—The Mean Arterial Pressure (MAP) Challenge

Cerebral pressure autoregulation, both static and dynamic, can be severely impaired following TBI [45]. The assessment of dynamic pressure autoregulation requires special equipment that may not be available to most ICUs. Static pressure autoregulation (sPAR), on the other hand, can be evaluated at the patient’s bedside [46,47]. In some cases, there may be hints that sPAR is intact. For example, the ICP may rise acutely following a MAP drop, and may recede when the MAP is restored with the administration of fluids or vasopressors. The consensus working group in the recent guidelines, suggest Rosenthal’s method for evaluating sPAR by using the MAP challenge [48,49]. In cases where autoregulation is preserved, and the baseline CPP is above the lower breakpoint of sPAR, a further rise of the CPP will result in vasoconstriction. Vasoconstriction will lead to decreased cerebral blood flow (CBF) and cerebral blood volume, and to a drop in the ICP. To perform the assessment, clinicians need to maintain vasopressor and sedatives infusions, as well as ventilation parameters stable. After recording the baseline MAP and CPP, the vasopressors are titrated to a MAP rise of 10 mmHg, and the patient’s response is observed for a maximum of 20 min. The ideal positive response comprises an ICP drop in response to the MAP rise [45,50]. Subsequently, the MAP and CPP need to be adjusted accordingly.

The assessment of sPAR can be associated with several clinical problems. Clinical experience is required to treat a possible spike of the ICP caused by the rising MAP in cases where the sPAR is disrupted. Furthermore, adjusting the MAP/CPP, is a separate clinical decision that needs to take into account the relative risks of increasing the vasopressor infusion rate. This may be difficult in trauma patients who require high vasopressor dose and/or have concurrent ARDS or cardiac dysfunction [51]. Finally, the sPAR status may not be stable over the clinical course of the patient, and may require frequent reassessment [50]. Several additional tools have been proposed in the assessment of CBF and sPAR, such as transcranial color duplex ultrasonography, near-infrared spectroscopy, and brain perfusion imaging [46,47,52,53].

## 3. Tier-Three Therapies

### 3.1. Therapeutic Hypothermia

For many years, therapeutic hypothermia was used for the management of ICH in TBI based on favorable findings derived mainly from experimental studies of ischemic models, according to which hypothermia has significant neuroprotective effects [54]. Lowering the body temperature, and in particular the brain temperature, below 36 °C, decreases the metabolic demands of the brain tissue, hence decreases the CBF, the cerebral blood volume, and the ICP. At the cellular level, hypothermia mitigates calcium induced neurotoxicity, neuronal apoptosis, inflammatory response, and cytotoxic edema [55]. Nevertheless, these experimental findings did not translate to positive clinical outcomes. In a meta-analysis of 18 trials published in 2016, no decrease in mortality was observed, while hypothermia was related to increased risk of pneumonia and cardiovascular complications [54,56]. The EUROTHERM study, a randomized trial that finally included 387 TBI patients, was discontinued due to safety concerns, because of worse outcomes in the hypothermia arm that were attributed to adverse reactions during the rewarming period. Interestingly, the control arm received higher-tier therapies and more barbiturates than the patients treated with hypothermia [57]. The POLAR study, another randomized trial using early, sustained prophylactic hypothermia in patients with severe TBI, did not show any improvement in outcomes, either. However, there were deviations from the cooling protocol that may have masked a possible beneficial effect of hypothermia [58]. Based on these results, current recommendations suggest the use of mild hypothermia, targeting core body temperatures of 35–36 °C as a tier-three intervention. Temperatures of< 35 °C are not recommended, due to increased risk for systemic complications [48]. In view of these facts, the term “targeted temperature management” (TTM), that reflects the currently recommended practice for post-cardiac arrest patients, would also be appropriate for TBI [55].

When considering TTM, the overall patient’s condition needs to be evaluated in view of anticipated side effects. The latter may include impaired cardiac contractility, coagulation and platelet dysfunction, increased risk for arrhythmias and infections, and significant fluid and electrolyte shifts [59]. These complications have been reported mainly in patients cooled down to 32–35 °C, and are more pronounced during the rewarming phase [57]. The targeted temperatures can be achieved with the use of external cooling measures. Cooling blankets or other devices with feedback control are appropriate when available, in order to avoid unwanted temperature shifts or body temperature below the desired level [59]. Notably, some patients are spontaneously hypothermic following TBI [60]. While there are no recommendations on temperature correction in this setting, maintaining temperatures at 35–36 °C and avoiding rewarming beyond this point seems reasonable [60]. Compared to other tier-three treatments, temperature management may be more suitable for patients without active bleeding and signs of shock, who are not candidates for surgical decompression.

### 3.2. Metabolic Suppression with Barbiturates

Pentobarbital and thiopentone are the most commonly used barbiturates for ICH in TBI. Both drugs are potent sedatives that can induce greater metabolic suppression than midazolam or propofol, and also have antiepileptic properties. By depressing brain function, oxygen consumption and metabolism, barbiturates also reduce the CBF and the ICP [3]. Barbiturates bind to neuronal γ-aminobutyric acid alpha (GABA_A_) receptors and cause neuronal hyperpolarization and inhibition of the action potential [61]. In addition, it has been shown that they reduce lactate and pyruvate production in the brain and inhibit lipid peroxidation mediated by free radicals [62]. These findings imply that barbiturates may also possess significant neuroprotective effects. Nevertheless, there is no evidence to support improvement in clinical outcomes with their use [63].

A plausible argument for this discrepancy is the association of barbiturates with significant side effects, the most prominent being hemodynamic compromise presenting as hypotension and myocardial depression. Consequently, the use of barbiturates in trauma patients with shock or myocardial injury is limited, since they may further increase vasopressor requirements for the maintenance of adequate CPP [64,65]. Other side effects are immunosuppression, hepatic and renal dysfunction, suppression of gut motility, and dyskalemias, which usually appear as hypokalemia during the loading phase and hyperkalemia during withdrawal. The latter may require renal replacement therapy in some cases [66,67]. At increased doses, barbiturates suppress the pupillary light reflex, in which case patient monitoring relies mainly on invasive measures or imaging. Finally, barbiturate use can lead to prolonged sedation due to drug accumulation, and consequently to prolonged need for mechanical ventilation [68,69,70,71,72].

When metabolic suppression with barbiturates is planned, it is advised to administer a test dose and record the patient’s response. A favorable response is characterized by ICP drop and concurrent maintenance of adequate CPP. If this is achieved, then loading doses can be administered, e.g., thiopental 250 mg boluses up to a total dose of 3–5 g, and then continuous infusion of 3–8 mg/kg/h. The endpoint of barbiturate administration is the control of the ICP with the minimum effective dose, in order to minimize side effects. This is achieved with the application of electroencephalographic monitoring and titration of the barbiturate infusion rate to a suppression–burst pattern of >>50%, since a further increase in barbiturate dose is unlikely to affect the ICP [73,74]. Other concurrent therapeutic goals include the maintenance of euvolemia and of the CPP [58]. Once ICH is treated and barbiturates are to be withdrawn, a gradual reduction over a few days is advised to avoid hyperkalemia and a rebound rise of the ICP.

While barbiturate use for metabolic suppression of the brain is suggested by guidelines, the so called low-dose (e.g., 2 mg/kg/h) barbiturate use has been adopted by many hospitals [68]. Adding low-dose barbiturates to other sedatives, such as midazolam and propofol, carries the risk of additive cardiovascular depression, but may also improve neuroprotection and reduce the time to drug elimination. Nevertheless, this practice is not supported by evidence and is not recommended by most experts in recent consensus guidelines [7]. A suppression burst pattern can also be induced by propofol and, in a small number of cases, by midazolam infusion, but none of these agents lowers the ICP as effectively as barbiturates. This is attributed to the profound suppression of cerebral rate of oxygen consumption during barbiturate induced suppression burst [43,75]. On the other hand, propofol carries significant risks when used in high doses and for prolonged periods (>5 mg/kg/h, >48 h). Cardiovascular depression, that may affect the MAP and CPP is commonly reported. Other side effects include the elevation of pancreatic enzymes, pancreatitis, lipemia, increased caloric administration due to the lipid formulation of the infused drug, and the propofol infusion syndrome (PRIS). PRIS is characterized by lactic acidosis, electrocardiographic changes, such as J-point and ST-segment elevation and T-wave inversion, hepatomegaly, and elevated transaminases. It can progress to rhabdomyolysis, renal failure, hyperkalemia, malignant arrhythmias, and cardiovascular collapse. Although PRIS is rare, the reported mortality rate is high (48–52%). The exact pathophysiology of PRIS is unclear, but possible mechanisms involve interaction of propofol with mitochondrial function and lipid metabolism [76].

### 3.3. Decompressive Craniectomy

The last modality in tier three is the physiologically most effective one, i.e., the surgical opening of the cranial cavity. Decompressive craniectomy lowers the ICP, and this has been confirmed by two randomized trials, the DECRA, published in 2011 and the RESCUE-ICP, published in 2016. There are many differences between these two studies. Patients in the DECRA trial were recruited early, within 72 h of TBI, and had a lower ICP threshold and lower-tier treatments applied [77]. Patients in the RESCUE ICP trial had an ICP of >25 mmHg, not responding to maximum medical therapy, including hypothermia, but not barbiturate coma [78]. In the DECRA trial, similar mortality was observed in the two study groups, with higher rates of unfavorable outcomes in the craniectomy group. In the RESCUE-ICP trial, there was a survival advantage in the intervention group. While there were higher rates of unfavorable outcomes among survivors at six-month follow-up, there were also statistically significant higher rates of favorable outcomes at 12 months [78].

Complications of decompressive craniectomy include infections, intracranial hemorrhage, seizures, transcranial herniation, formation of subdural hygromas, and hydrocephalus. These, combined with the risks of cranioplasty [49] and of a poor neurological outcome, may hinder the decision for craniectomy. The risks described, as well as the probable need for long-term care, have to be thoroughly explained to the patients’ families before the procedure. Patients with acute subdural hematomas often have concomitant underlying intraparenchymal hematomas and contusions [79]. In many cases where subdural hematomas warrant evacuation, the brain bulges beyond the table of the skull postoperatively, and there is a surgical option for primary craniectomy, i.e., “to leave the bone flap out”. While there is limited evidence in support of primary craniectomy in this setting, there are some studies that show better outcomes with this strategy [80,81] and a relevant randomized trial is ongoing [82]. In the meantime, decompressive craniectomy remains a tier-three therapy, from which previously fit patients with unilateral pathology, and adequate medical and social support are more likely to benefit. Decompressive craniectomy also remains a rescue option for patients not responding to conservative treatments [49].

## 4. Second- and Third-Tier Therapies in Low- and Middle-Income Countries (LMICs)

**Figure 3:**
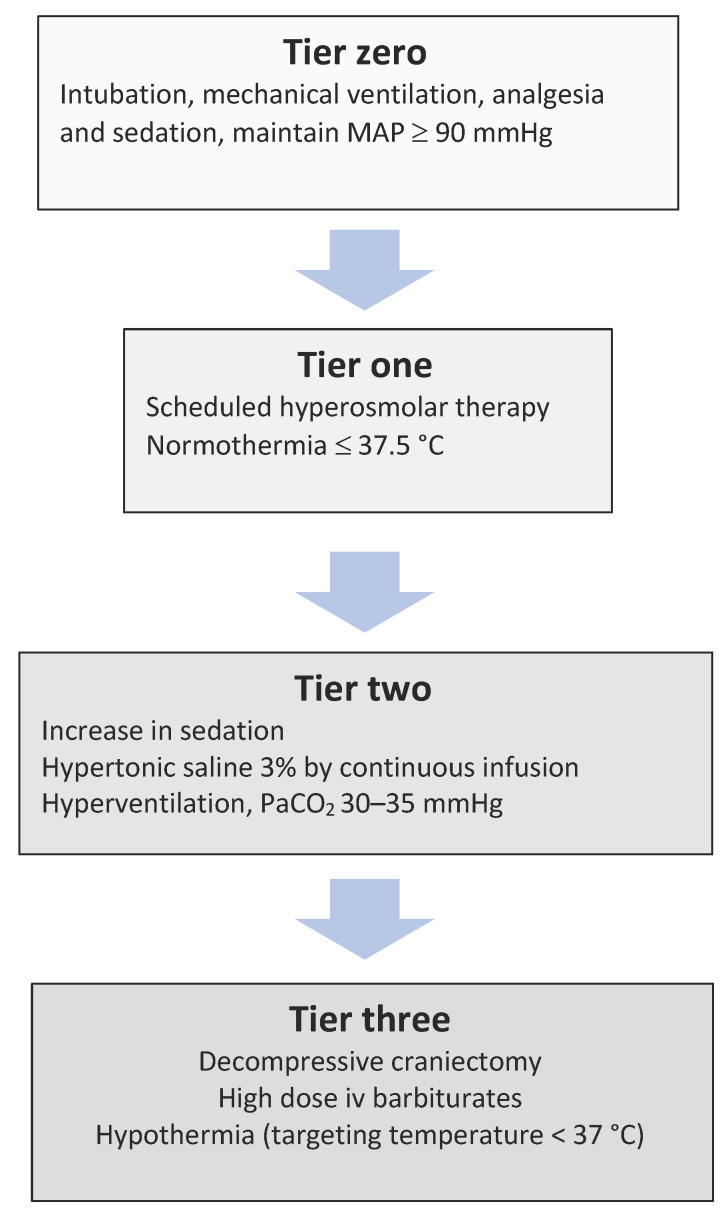
A simplified version of the three-tier therapy protocol proposed for the treatment of suspected intracranial hypertension when intracranial pressure monitoring is not employed. Decisions on escalation and de-escalation are based on serial clinical examination and computerized tomography imaging. MAP: mean arterial pressure; PaCO_2_: partial arterial pressure of carbon dioxide.

The management of ICH in TBI patients is even more challenging in developing countries. Surgical procedures may have a more central role in the management of ICH, because available resources for neuromonitoring are limited, as opposed to the capacity for surgical decompression. Following the BEST TRIP (the Benchmark Evidence from South American Trials: Treatment of Intracranial Pressure) trial [83], protocols such as the CREVICHE (Consensus REVised ICE algorithm) have appeared, which give directions on the application of tiered therapies in this setting, based on clinical examination and brain CT findings [84]. Figure 3 depicts a simplified version of the protocols suggested in low-income countries setting, when ICP monitoring is not applied. Scheduled hyperosmolar therapy as well as continuous infusion of 3% hypertonic saline are allowed in this setting, and decompressive craniectomy remains a tier-three intervention [84]. While most craniectomies performed in low/middle-income countries are primary procedures, the clinical decisions, particularly for secondary craniectomies, may be difficult, due to the limited capability for long-term support and rehabilitation, that may compromise neurological outcomes. The proposed surgical techniques are also discussed in the relevant literature. Floating or hinged bone flaps have been suggested to avoid the need for reconstructive cranioplasty, but there are no recommendations to support this practice. Current guidelines advise that these procedures should be performed by neurosurgeons or at least neurosurgical trainees that are adequately trained, and that decisions should be made in the context of local knowledge, medical resources, capacity for long-term care, and cultural beliefs [79,85].

## 5. Conclusions

Tier-two and -three therapies for ICH in TBI are associated with significant adverse effects and complications, and are applied when ICH poses a bigger threat to the patient. The tier concept allows for flexibility in the therapeutic choices and for patient-tailored strategies in different resource settings. Individualized approaches can be achieved by the use of advanced imaging and neuromonitoring modalities. Future research is oriented towards strengthening the existing evidence and identifying the specific patient profiles that can benefit from each separate therapeutic modality.
